# Assessment of coronary microvascular resistance in the chronic infarcted pig heart

**DOI:** 10.1111/jcmm.12089

**Published:** 2013-08-03

**Authors:** Stefan Koudstaal, Sanne J Jansen of Lorkeers, Frebus J van Slochteren, Tycho IG van der Spoel, Tim P van de Hoef, Joost P Sluijter, Maria Siebes, Pieter A Doevendans, Jan J Piek, Steven AJ Chamuleau

**Affiliations:** a Department of Cardiology Division Heart and Lungs, University Medical Center UtrechtUtrecht, The Netherlands; b Interuniversity Cardiology Institute of the Netherlands (ICIN)Utrecht, The Netherlands; c Department of Cardiology, Academic Medical CenterAmsterdam, The Netherlands; d Department of Biomedical Engineering and Physics, Academic Medical CenterAmsterdam, The Netherlands

**Keywords:** Coronary microvascular resistance, Capillary density, Angiogenesis, Chronic MI

## Abstract

Pre-clinical studies aimed at treating ischemic heart disease (*i.e*. stem cell- and growth factor therapy) often consider restoration of the impaired microvascular circulation as an important treatment goal. However, serial *in vivo* measurement hereof is often lacking. The purpose of this study was to evaluate the applicability of intracoronary pressure and flow velocity as a measure of microvascular resistance in a large animal model of chronic myocardial infarction (MI). Myocardial infarction was induced in Dalland Landrace pigs (*n* = 13; 68.9 ± 4.1 kg) by a 75-min. balloon occlusion of the left circumflex artery (LCX). Intracoronary pressure and flow velocity parameters were measured simultaneously at rest and during adenosine-induced hyperemia, using the Combowire (Volcano) before and 4 weeks after MI. Various pressure- and/or flow-derived indices were evaluated. Hyperemic microvascular resistance (HMR) was significantly increased by 28% in the infarct-related artery, based on a significantly decreased peak average peak flow velocity (pAPV) by 20% at 4 weeks post-MI (*P* = 0.03). Capillary density in the infarct zone was decreased compared to the remote area (658 ± 207/mm^2^ *versus* 1650 ± 304/mm^2^, *P* = 0.017). In addition, arterioles in the infarct zone showed excessive thickening of the alpha smooth muscle actin (αSMA) positive cell layer compared to the remote area (33.55 ± 4.25 μm *versus* 14.64 ± 1.39 μm, *P* = 0.002). Intracoronary measurement of HMR successfully detected increased microvascular resistance that might be caused by the loss of capillaries and arteriolar remodelling in the chronic infarcted pig heart. Thus, HMR may serve as a novel outcome measure in pre-clinical studies for serial assessment of microvascular circulation.

## Introduction

Coronary artery disease is a major cause of mortality and morbidity worldwide that can be held responsible for 7 million deaths annually [1]. Myocardial ischemia is associated with a poor prognosis and could give rise to disabling complaints of refractory angina pectoris [2]. The concept of restoration of impaired blood flow by the formation of new capillaries (angiogenesis) to treat ischemia in tissue has a high scientific appeal [3]. Therefore, numerous broadly ranging strategies to promote angiogenesis (*e.g*. stem cell therapy, growth factor delivery and microRNA interference) are currently being explored in the pre-clinical setting [4–7]. Serial *in vivo* assessment of the status of the myocardial microcirculation remains cumbersome [8]. Thus, angiogenesis is often reported based on *ex vivo* histologic analysis of the area of interest.

Several intracoronary pressure- and flow velocity-derived indices have been studied for the ability to draw inferences on the healthy or diseased status of the coronary circulation. Among these indices, there is the clinically widely used fractional flow reserve (FFR), based on intracoronary pressure, to steer clinical decision-making in epicardial stenoses [9, 10]. Coronary flow velocity reserve (CFVR), derived from intracoronary flow velocities, represents the ability to increase coronary flow under hyperemic conditions. Unfortunately, CFVR varies between and within patients as it depends on several parameters such as metabolic demand, the diastolic time fraction, blood pressure and microvascular disease [11, 12]. Relative flow velocity reserve, the ratio of CFR in the stenosed and healthy coronary artery, has been proposed as an alternative, but did not lead to clinical application [13]. An alternative method to assess the functioning of myocardial vasculature is by pressure- and flow velocity-derived microvascular resistance [14]. This has become possible by the combination of simultaneously measured pressure and flow velocity, to yield an index referred to as hyperemic microvascular resistance (HMR) [15, 16].

We suggest that elevated microvascular resistance could serve as a novel outcome measure for pre-clinical studies that investigate novel treatment strategies to restore ischemia in myocardial tissue by means of arteriogenesis and/or angiogenesis. The aim of this study was to investigate the effect of chronic myocardial infarction (MI) in a large animal model on microvascular resistance and to study the potential underlying mechanisms reflecting this parameter.

## Materials and methods

### Animals and study design

Thirteen 6-month-old female Dalland Landrace pigs (weighing 69 ± 4 kg) received care in accordance with the *Guide for the Care and Use of Laboratory Pigs* prepared by the Institute of Laboratory Animal Resources. Experiments were approved by the Animal Experimentation Committee of the Medicine Faculty of the Utrecht University, the Netherlands. First, intracoronary pressure and flow velocity and pressure volume (PV) loop analysis was measured in healthy animals. Next, these animals were subjected to MI, induced by a 75-min. balloon occlusion of the left circumflex artery (LCX). Four weeks after the MI, functional end-point analysis was repeated. The schematic study design is shown in Figure S2.

### Myocardial infarction

The MI was induced as previously described [17]. Briefly, animals were sedated and general anesthesia was maintained by continuous infusion of midazolam (0.7 mg/kg/hr), sufentanil citrate (6 μg/kg/hr) and pancurorium bromide (0.1 mg/kg/hr) *via* the canulated ear vein. The animals were mechanically ventilated with a positive pressure ventilator (FiO_2_ 0.50) under continuous capnography.

Arterial access was achieved by canulating the internal carotid artery and MI was induced by a 75-min. balloon occlusion of the proximal LCX. Prior to the infarction, a bolus of amiodarone (300 mg) and metoprolol (5 mg) was infused intravenously in 45 min. to minimize onset of cardiac arrhythmias.

### Intracoronary pressure and flow velocity assessment

Intracoronary pressure and flow velocity were measured simultaneously by using the Combowire® (Volcano Corporation, San Diego, CA, USA) as previously described [15, 18]. Pressure and flow velocity signals, combined with aortic pressure and ECG signals were recorded using the ComboMap® system (Volcano Corporation). Intracoronary pressure and flow velocity were assessed prior to the infarction and 4 weeks after MI in the infarct-related artery (LCX) and the reference artery (LAD). Nitroglycerin (200 mcg) was injected intracoronarily to prevent coronary spasms. Next, the Combowire was placed in the proximal section of the LCX and the LAD. Velocity and pressure signals were recorded during rest and peak hyperemia. Hyperemia was induced by intracoronary bolus of 60 mcg adenosine. At least three representative measurements were performed per vessel.

### Analysis of pressure- flow velocity-derived indices

Data sets were stored digitally and analysed offline using AMC Studymanager, a custom software package (written in Delphi *versus* 6.0, Borland Software Corporation and Delphi *versus* 2010, Embarcadero, San Francisco, CA, USA). CFVR was calculated as CFVR = pAPV/bAPV, where APV is average peak flow velocity in cm/s. The bAPV and pAPV were calculated as the mean of four beats at rest and the mean of three successive beats with the highest flow velocity respectively. Hyperemic microvascular resistance was calculated as HMR = P_d_/pAPV, where both P_d_ and pAVP were derived from the mean of three beats at hyperemia [19].

### Pressure–Volume loop protocol

Pressure–volume loops were acquired using a 7-F conductance catheter that was placed in the left ventricle. The catheter was connected with a signal processor (Leycom CFL, Zoetermeer, the Netherlands). Data were collected during steady-state conditions with the respirator system turned of at end-expiration. Data analysis and calculation were performed on custom-made software (CD Leycom, Zoetermeer, the Netherlands).

### Histology

Four animals, that served as control treated animals in a larger study [20], were killed 8 weeks after MI by exsanguination under general anesthesia (see Figure S2). After excision of the heart, the left ventricle was cut into five slices from base to apex and incubated in 1% triphenyl-tetrazolium chloride dissolved in phosphatase buffered saline (PBS) at 37°C for 15 min. Next, the slices were washed in PBS and photographed digitally (Sony Alfa 55). Snap frozen tissue samples from the infarct zone and remote area (septal wall) were embedded in Tissue-Tek (Sakura, Torrance, CA, USA) and 7 μm cryosections were prepared on a microtome (Leica, Buffalo Grove, IL, USA). Sections were dried for 30 min. at room temperature (RT) and fixed in acetone. Subsequently, slides were incubated with 0.1% Triton X-100 (Sigma-Aldrich, St. Louis, MO, USA) in PBS with 1% bovine serum albumin (BSA), blocked in 10% goat serum, incubated overnight at 4°C in 1% goat serum with primary antibodies against α-smooth muscle actin (αSMA) (1:1500, Mouse monoclonal, Clone 1A4, Sigma-Aldrich) and CD31 (1:100, rabbit polyclonal, Abcam, Cambridge, MA, USA) and then incubated for 1 hr at RT with secondary antibodies (Invitrogen, Grand Island, NY, USA). Slides were mounted in Fluoromount (Southern Biotech, Birmingham, AL, USA) and fluorescence images were acquired on an Olympus DP71 microscope. For image analysis, the number of arterioles (defined as aSMA-positive vessels >20 μm and <300 μm) and wall thickness (defined as the wall thickness of the aSMA-positive cell layer) was measured in 10 different fields/section in the infarct zone and the border zone at ×40 magnification using ImageJ (1.44 g). The number of arterioles was expressed per 1.0 mm^2^.

The density of capillaries in the infarct region and remote area was assessed by staining with an antibody against CD31 (1:100, mouse monoclonal, AbD Serotec, Raleigh, NC, USA). Endogenous peroxidase in the cryosections was blocked with 0.15% hydrogen peroxide in acetone for 15 min. at RT. The 2° antibody used was horse antimouse, biotinylated (1:200; Invitrogen) for 60 min. at RT followed by a streptavidin-HRPO in PBS (1:1000) for 60 min. at RT. The chromogen 3-amino-9-ethylcarbazole (AEC; Sigma-Aldrich) was used to visualize capillaries. The slides were counterstained with hematoxylin for identification of nuclei. The number of capillaries (defined as one to three cells spanning the CD31-positive vessel circumference) was determined by counting 10 fields/section in the infarct zone and the peri-infarct border zone at ×40 magnification. The number of capillaries was expressed per 1.0 mm^2^.

### Statistics

Continuous variables are presented as mean ± SD. Analysis was performed by paired or two sample *t*-test. The assumption that the variable must be normally distributed was checked by QQ plots and the Kolmogorov-Smirnov test. All tests were performed using SPSS Statistics 17.0. Values of *P* < 0.05 were considered statistically significant.

## Results

### Effect of chronic MI on coronary pressure and flow velocity

**Fig. 1:**
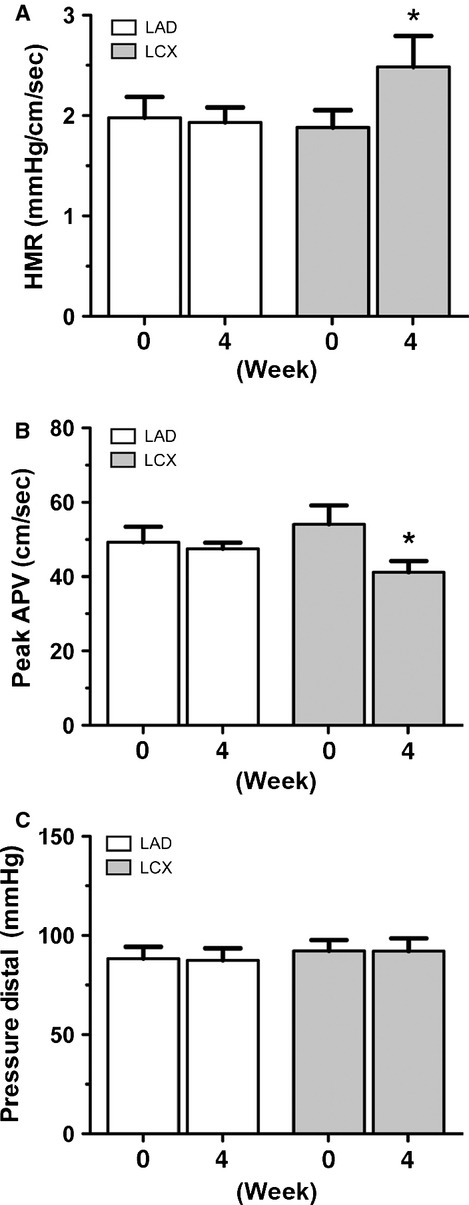
Coronary pressure-/flow derived assessment of microvascular circulation. (**A**) Combined pressure and peak hyperemic flow were used to calculate hyperemic microvascular resistance (HMR) in both the reference artery (white bars) and the infarct-related artery (grey bars). Four weeks after myocardial infarction (MI), HMR in the left circumflex artery (LCX) was increased (* denotes *P* = 0.03). (**B**) The peak APV was decreased in the LCX at 4 weeks after MI compared to baseline (* denotes *P* = 0.05). (**C**) Intracoronary pressure measured by the Combowire did not change throughout the study. Error bars represent SEM.

The mean coronary diameter at baseline was 3.3 ± 0.7 mm and 3.1 ± 0.8 mm in the LCX and LAD, respectively, and did not change over time. Coronary pressure- and flow velocity-derived indices at baseline and follow-up are shown in Table 1. Hyperemic microvascular resistance in the infarct-related artery (LCX) was significantly higher at follow up (Fig. 1A; 2.4 ± 1.1 mmHg/cm/sec.) compared to baseline (Fig. 1A; 1.9 ± 0.6 mmHg/cm/sec.; *P* = 0.03), indicating an increase in regional microvascular resistance in the area of interest. The increased HMR in the LCX was based on an impaired peak coronary flow response to distal vasodilation at 4 weeks compared to baseline (Fig. 1B; 42.5 ± 11.4 cm/sec. *versus* 53.0 ± 17.3 cm/sec., respectively; *P* = 0.05). The distal intracoronary pressure did not show any change between 4 weeks after MI and baseline (Fig. 1C; 92.4 ± 21.2 mmHg *versus* 92.4 ± 19.1 mmHg; *P* = 0.50). Coronary flow velocity at rest was also impaired 4 weeks after MI compared to baseline (Figure S1). As a result, the CFVR remained unchanged following MI compared to baseline values (Figure S1). The reference artery (LAD) did not show any change in coronary pressure and velocity-derived indices (Fig. 1A–C), indicating a stable and unchanged ability to regulate flow velocities to myocardial demand.

**Table 1:** Parameters before and 4 weeks after MI

	Baseline	4 weeks	Difference		
	Mean ± SD	Mean ± SD	Mean	%	Sign.
MAP (mmHg)	95.7 ± 20.1	96.4 ± 20.6	0.67	0.7%	0.91^a^
HR (b/min.)	64.4 ± 10.1	57.0 ± 12.9	−7.36	−11.4%	0.16^a^
EF (%) (*n* = 12)	65.7 ± 6.7	55.3 ± 8.5	−10.13	−15.4%	0.00^c^
Weight (kg)	68.9 ± 4.1	72.3 ± 4.0	3.42	5.0%	0.01^a^
Infarct-related artery (LCX) *n* = 13					
FFR	1.0 ± 0.0	1.0 ± 0.0	0.01	0.9%	0.83^b^
CFVR	2.9 ± 0.4	3.0 ± 0.7	0.02	0.7%	0.46^c^
bAPV (cm/sec.)	18.0 ± 4.2	14.9 ± 4.0	−3.17	−17.6%	0.03^c^
pAPV (cm/sec.)	53.0 ± 17.3	42.5 ± 11.4	−10.46	−19.7%	0.05^c^
Pd (mmHg)	92.4 ± 19.1	92.4 ± 21.2	0.00	0.0%	0.50^c^
HMR (mmHg/cm/sec.)	1.9 ± 0.6	2.4 ± 1.1	0.53	28.4%	0.03^c^
Reference artery (LAD) *n* = 12					
FFR	1.0 ± 0.0	1.0 ± 0.0	0.00	0.5%	0.53^b^
CFVR	2.8 ± 0.5	3.0 ± 0.5	0.13	4.8%	0.21^c^
bAPV (cm/sec.)	17.3 ± 3.0	16.5 ± 2.9	−0.79	−4.5%	0.25^c^
pAPV (cm/sec.)	50.0 ± 13.3	47.7 ± 5.2	−2.25	−4.5%	0.25^c^
Pd (mmHg)	88.9 ± 19.8	87.9 ± 20.7	−1.00	−1.1%	0.42^c^
HMR (mmHg/cm/sec.)	1.9 ± 0.7	1.9 ± 0.5	−0.04	−1.9%	0.42^c^

a Two-tailed paired T-test.

b Wilcoxon Signed Ranks test.

c One-tailed paired T-test.

T = 0: before MI; T = 4 week: 4 weeks after MI; MAP: Mean aortic pressure; HR: heart rate; EF: LV ejection fraction; CK-MB ratio: ratio of CK-MB before ischemia and 30 min. after reperfusion; FFR: Pd/aortic pressure; CFVR: pAPV/bAPV; Pd: intracoronary pressure; HMR: Pd/pAPV.

### The effect of decreased vascular density of the scar tissue on microvascular resistance

**Fig. 2:**
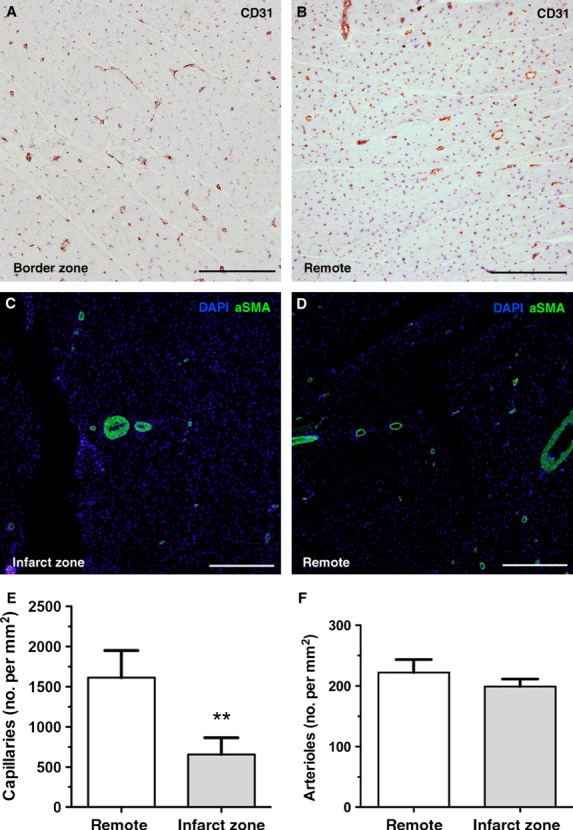
Altered vascular density of the scar tissue impairs microvascular resistance. Representative photographs showing microscopic fields of transversally oriented cardiomyocytes with CD-31 positive capillaries (**A** and **B**) or immunofluorescent-labelled αSMA (**C** and **D**). (**A**) Peri-infarct zone in the left circumflex artery (LCX) vascularized area shows a decreased number of CD-31 positive capillaries compared to (**B**) the remote area of the LAD (**C** and **D**). The number of αSMA positive arterioles is not different between infarct zone and remote area. Quantification for (**E**) CD31 positive capillaries (** denotes *P* = 0.0009) and (**F**) αSMA+ arterioles (remote *versus* infarct zone; *P* = 0.366). All scale bars represent 500 μm; error bars represent SEM.

To examine the role of myocardial vascular density in microvascular resistance, we quantified the CD31+ capillaries (Fig. 2A and B) and the αSMA+ positive arterioles (Fig. 2C and D) in both the infarct area and the remote area. The number of CD31+ capillaries was reduced in the scar tissue (Fig. 2E; 658 ± 207/mm^2^) compared to the remote area (Fig. 2E; 1650 ± 304/mm^2^, *P* = 0.0009). Regarding the number of arterioles, there was no change in the infarct area (Fig. 2F; 199 ± 30/mm^2^) compared to the remote area (Fig. 2F; 222 ± 52/mm^2^, *P* = 0.369). Thus, these results indicate that the balance between capillaries and larger arterioles is shifted in the infarct scar on the basis of a decreased number of capillaries per number of arterioles.

### Adverse remodelling of αSMA+ arterioles and changes in extracellular matrix composition

**Fig. 3:**
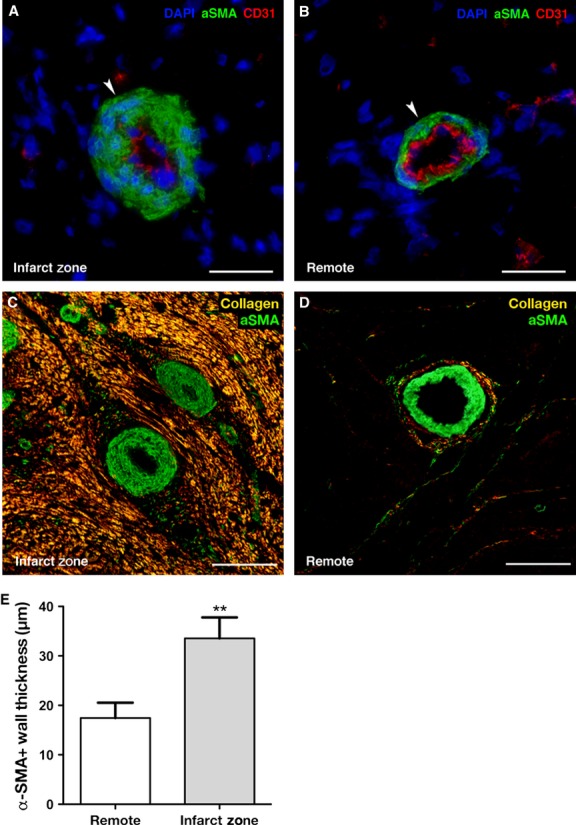
Structural changes in arterioles and extracellular matrix increase microvascular resistance. Representative photographs showing microscopic fields of co-staining with immunofluorescent-labelled αSMA (green signal), CD-31 (red signal) and nuclei counterstained with 4′,6-diamino-2-phenylindole (DAPI). (**A**) In the infarct zone, there is pronounced thickening of the αSMA + cells of the arterioles. (**B**) This phenomenon did not occur in arterioles from samples of the LV remote area. (**C**) In the infarct zone αSMA+ arterioles are embedded within dense collagen fibres (yellow signal). In contrast, (**D**) the remote area displayed small amounts of collagen dispersed in between patches of viable cardiomyocytes and constituted a small rim surrounding the arterioles. (**E**) Quantification of αSMA+ wall thickness shows a twofold increase (** denotes *P* = 0.002). All scale bars represent 50 μm; error bars represent SEM.

Next, we examined the morphology of the αSMA+ arterioles to elucidate the role of adverse remodelling as a mechanism for impaired coronary response to hyperemia. Strikingly, the αSMA+ arterioles within the infarct zone were characterized by a pronounced thickening of the αSMA+ cells forming a dense layer surrounding the CD31+ endothelial cells compared to the remote area where αSMA+ cells appeared as a small rim surrounding the arterioles (Fig. 3A–B). The average thickness of the αSMA+ cells per arteriole was increased in the infarct area (Fig. 3E; 36 ± 14 μm) compared to the remote area (Fig. 3E; 15 ± 4 μm; *P* = 0.002).

Furthermore, when combining the αSMA+ fluorescent images with the polarized light microscopy of picrosirius stained collagen, arterioles appeared entrapped in between the dense collagen fibres (Fig. 3C) in the infarct zone. In contrast, the remote area revealed a small layer of collagen surrounding the arterioles dispersed in between the viable myocardium (Fig. 3D). Altogether, these findings indicate that both adverse remodelling of the αSMA+ vessels and altered extracellular matrix composition could be potential underlying mechanisms leading to impaired coronary flow response and increased HMR.

## Discussion

Since 1990s, intracoronary pressure and flow velocity measured with sensor-tip guidewires have been introduced as a novel approach for assessment of coronary hemodynamics [21]. The use of guidewire-based assessment of coronary hemodynamics in the cardiac catheterization laboratory is well-established to guide clinical decision-making [15]. Yet, this powerful tool has thus far not been implemented as a functional end-point in large animal models of ischemic heart disease and in particular those designed to validate new angiogenic therapies such as growth factors/cytokines, stem cell therapy or gene therapy.

Here, we have shown that intracoronary pressure-/flow velocity could successfully detect an increased hyperemic microcirculatory resistance in a porcine model of chronic MI. Potential mechanisms pertaining to this increased microvascular resistance are threefold. First, capillary density in the infarct scar tissue was reduced twofold while larger arterioles were present in similar numbers as in the remote area. Second, arterioles within the infarct zone were characterized by a marked thickening of the αSMA+ cell layer. Third, infarct zone arterioles were dispersed in between dense collagen fibres that could hamper the dilatory capacity of these vessels to hyperemic stimuli. Collectively, these data indicate a dual origin for the increase in HMR, both on the level of arterioles as well as on the level of capillaries.

### The role of capillaries in microvascular dysfunction

From previous data in dogs, Jayaweera and coworkers showed that, in the absence of a stenosis or hyperemia, vasoconstriction of the arterioles constitute the largest resistance (61 ± 5%) of total myocardial vascular resistance on coronary flow, compared to the capillary (25 ± 5%) or venous (14 ± 4) compartments [22]. Study by Friedman *et al*. confirmed the modest role of capillaries at rest by showing that increased flow recruited to opening of additional capillaries thereby facilitating the additional flow [23]. Due to the redistribution of coronary resistance, the capillary compartment had the highest vascular resistance at maximal vasodilation, accounting for 75% of the total myocardial vascular resistance [22]. Thus, the observed reduction in capillary density in the infarct zone and its effect on the increase in HMR support the notion that the capillary compartment plays a crucial role during a hyperemic response of the coronary flow. The mean capillary density of 1650 ± 304/mm^2^ in the healthy porcine myocardium is in reasonable agreement with the previously reported 1956 ± 231/mm^2^ in the human heart [24]. More importantly, we observed a capillary density in the porcine infarct scar area of 658 ± 207/mm^2^, which mimicked reported values of human ischemic cardiomyopathy of 1124 ± 226/mm^2^ by Karch [24] and 771 ± 68/mm^2^ by Mehrabi [25] in explanted hearts from patients with ischemic cardiomyopathy undergoing heart transplantation.

### Microvascular dysfunction and arteriolar remodelling in the infarct zone

Another finding of the present study is the contribution of increased arteriolar wall thickness on the curtailed coronary flow. This phenomenon has been observed previously in patients with arterial hypertension [26]. Furthermore, although in a distinct patient population, increased arteriolar wall thickness in heart allografts was linearly associated with an increase in HMR [27]. Coronary arterioles tend to structurally adapt to a wide variety of pathophysiological situations. In pigs that underwent a gradual coronary stenosis, a range of structural changes and impaired response to bradykinin-1 in arterioles distant to a stenosis was observed [28]. Additionally, type-2 diabetes induced similar narrowing of coronary arteriolar lumen by increase in wall thickness [29]. Given the role of arteriolar dilation during hyperemic peak flow, it is conceivable that this arteriolar remodelling negatively affects their ability to relax and hence impair their vasodilatory capacity and thereby increases vascular resistance.

### The effect of the extracellular matrix on the microvascular resistance

Besides the altered vasculature, change in extracellular matrix has also been shown to moderate the vascular ability to respond to different flow conditions [26]. In addition, Berry *et al*. showed that myocardial tissue after infarction is more rigid than healthy myocardium [30]. In line with these findings, we report an increased layer of dense collagen fibres in the close proximity of arterioles. Although the effect of fibrosis on arteriolar vasodilatation was not quantified in this study, we speculate that the increased collagen content further reduced the normal hyperemic flow response.

Taken together, these results provide support for the notion that HMR measured by the Combowire can play an important role in pre-clinical large animal models in which serial assessment of microvascular circulation is warranted.

## Study limitations

When extrapolating the current findings to an equivalent patient population of chronic MI, it should be kept in mind that several determinants of increased HMR have not been incorporated in this animal model, such as age, diabetes or reduced pressure due to atherosclerosis and/or subsequent formation of thrombus and/or thrombotic emboli- in the epicardial vessels [31].

Secondly, it should be borne in mind that the use of adenosine has a few practical limitations that precludes an unbiased estimate of the maximal coronary flow. We cannot rule out that dysfunction of vessels and thereby improper reaction on adenosine might also contribute to the increased HMR as well. In coronary artery disease and after MI, α-adrenergic vasoconstriction occurs and adenosine does not dissolve this α-adrenergic vasoconstriction [32].

Thirdly, regarding mechanisms of increased microvascular resistance, undoubtedly, numerous processes in the chronic infarct can account for an increase in microvascular resistance, such as mechanical stresses and strain of the microvessels during myocardial contraction and relaxation or neurologic and/or metabolic dysregulation on vascular alpha-tones.

Finally, morphometric analysis of arterioles may be influenced by fixation of the myocardial tissue. However, we did not observe any signs of arteriolar remodelling in the healthy myocardium, suggesting a dominant role for the chronic infarct as a causal factor.

## Supporting information

**Figure S1.** Assessment of coronary flow velocity reserve.

**Figure S2.** Experimental study design.

**Figure S3.** Infarct size.
