# Supplementary material for: Assessment of coronary microvascular resistance in the chronic infarcted pig heart
Source: J Cell Mol Med. 2013 Aug 3;17(9):1128–35. doi: 10.1111/jcmm.12089 (PMC4118172; doi:10.1111/jcmm.12089)
Supplement: Supplementary file 1 [file jcmm0017-1128-SD1.docx]

**ONLINE APPENDIX FOR THE FOLLOWING JCMM ARTICLE**

**TITLE:** Assessment of Coronary Microvascular Resistance in the Chronic Infarcted Pig Heart.

**AUTHORS:** S. Koudstaal, S.J. Jansen of Lorkeers, F.J. van Slochteren, T.I.G. van der Spoel*,* T.P. van de Hoef, J.P Sluijter, M. Siebes, P.A. Doevendans, J.J. Piek, S.A.J. Chamuleau

**Supplementary Information**

**
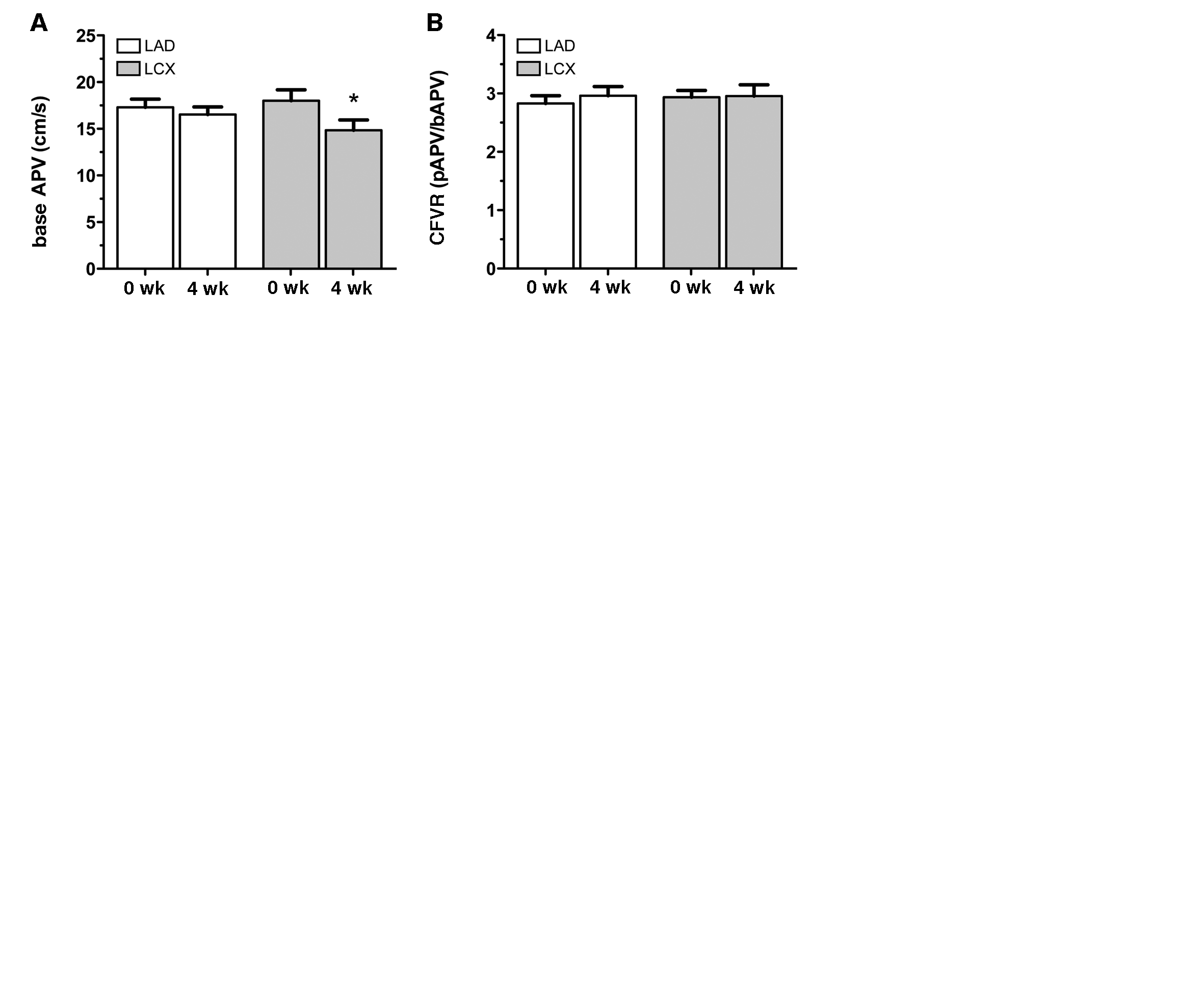
**

**Supplementary figure 1. Assessment of Coronary Flow Velocity Reserve**

**(A)** At four weeks follow up, coronary flow velocity under base condition was decreased. **(B)** As a result, the ratio between peak and base APV, reflected by the CFVR, remained similar throughout the follow up duration.

**Supplementary figure 2. Experimental Study Design**

**(A)** Schematic overview of the *in vivo* study design of a chronic MI model and different time points of intracoronary pressure and flow velocity analysis, namely before induction of MI (baseline) and at 1 month follow up. Histology is obtained at two months after MI in a subset of animals (n=4) which have been used as a negative control group described elsewhere.[[1](#_ENREF_1)]

**
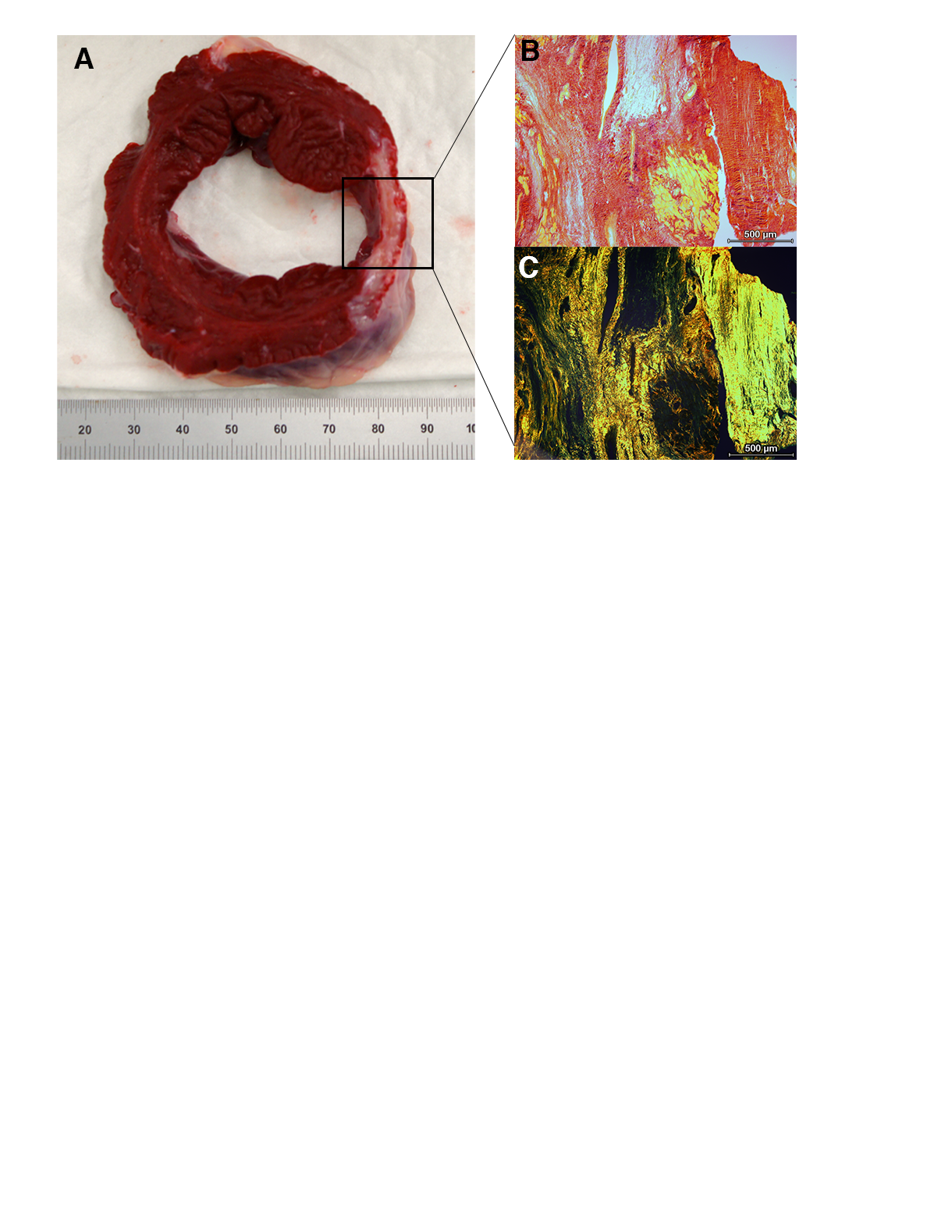
**

**Supplementary figure 3. Infarct size**

**(A)** Representative photograph of triphenyltetrazolium chloride (TTC) staining of the left ventricle at the level of the papillary muscle show varying forms of transmural infarct size (white color) in the viable myocardium (red color). **(B)** Picric red staining shows the infarct collagen (red signal) and viable myocardium (yellow signal). **(C)** Under polarized light, picric red staining for collagen is visible as a bright yellow signal.

**Supplementary figure 3. Dose-finding of intracoronary adenosine and peak hyperemia**

Average of three consecutive measurements of peak hyperemic flow in response to increasing doses of adenosine administered intracoronary. For further experiments, intracoronary admission of 60 mcg was set as the adenosine dose.

**References**

1. **Koudstaal S, Bastings MMC, Feyen D, Waring CD, Slochteren FJV, Dankers PYW, Torella D, Sluijter JPG, Nadal-Ginard B, Doevendans PA, Ellison GM, Chamuleau SA.** Sustained Delivery of Insulin-Like Growth Factor-1/Hepatocyte Growth Factor Stimulates Endogenous Cardiac Repair in the Chronic Infarcted Pig Heart. J Am Coll Cardiol. 2013;61(10_S): doi:10.1016/S0735-1097(13)61141-9
